# A novel diagnostic algorithm to predict significant liver inflammation in chronic hepatitis B virus infection patients with detectable HBV DNA and persistently normal alanine transaminase

**DOI:** 10.1038/s41598-018-33412-z

**Published:** 2018-10-18

**Authors:** Qiang Li, Yu Zhou, Chenlu Huang, Weixia Li, Liang Chen

**Affiliations:** 1 Department of Liver Disease, Shanghai Public Health Clinical Center, Fudan University, Shanghai, 201508 China; 2 Department of Infectious Disease, Ruian people’s hospital, Wenzhou, Zhejiang, 325200 China

## Abstract

Significant liver inflammation might be found in 20–34% of chronic hepatitis B virus (HBV) infection patients with detectable HBV DNA and persistently normal alanine transaminase (ALT) (PNALT). We aimed to develop a diagnostic algorithm to predict significant liver inflammation in these specific patients. Using liver biopsy as the gold standard, we developed a novel, simple diagnostic algorithm to predict significant liver inflammation in a training set of 365 chronic HBV infection patients with detectable HBV DNA and PNALT, and validated the diagnostic accuracy in a validation set of 164 similar patients. The novel algorithm (AAGP) attributed to age, ALT, gamma-glutamyl transpeptidase (GGT), and platelet count was developed. In the training set, the area under the receiver operating characteristic curve (AUROC) of AAGP was higher than that of ALT and aspartate transaminase (AST), to diagnose significant liver inflammation (0.77, 0.67, and 0.59, respectively, *p* < 0.001). In the validation set, the AUROC of AAGP was also higher than ALT and AST (0.75, 0.61, and 0.54, respectively, *p* < 0.001). Using AAGP ≥2, the sensitivity and negative predictive value (NPV) was 91% and 93%, respectively, to diagnose significant liver inflammation. Using AAGP ≥8, the specificity and NPV was 91% and 86%, respectively, for significant liver inflammation. In conclusion, the AAGP algorithm is a novel, simple, user-friendly algorithm for the diagnosis of significant liver inflammation in chronic HBV infection patients with detectable HBV DNA and PNALT.

## Introduction

Infection with hepatitis B virus (HBV) is a public health problem worldwide, and 240 million people estimated to experience chronic HBV infection^1^. In China, HBV infection is moderately endemic, and chronic hepatitis B (CHB) is the main cause of cirrhosis, liver de-compensation, and hepatocellular carcinoma (HCC)^2^. Among chronic HBV infection patients, those with significant liver inflammation have a much greater risk of cirrhosis, liver de-compensation, and HCC^3^. According to the guidelines for chronic HBV infection, it is critical to identify patients with significant liver inflammation, and treat them immediately^4–6^. Therefore, the diagnosis of significant liver inflammation is important for physicians to evaluate the prognosis and decide the treatment initiation of chronic HBV infection patients.

Liver biopsy is the gold standard for the diagnosis of liver inflammation. However, liver biopsy is an invasive procedure, carrying a risk of rare but potentially life-threatening complications^7^. In addition, the expenses for liver biopsy were high-this again limits the use of liver biopsy for mass screening. These limitations of liver biopsy have led to the development of non-invasive markers of liver inflammation. Biochemical test is usually used to diagnose liver inflammation because of its inexpensive and non-invasive advantages. The most commonly used biochemical test reflecting liver inflammation is serum alanine transaminase (ALT).

Generally, patients with significantly elevated ALT (>2 times upper limit of normal (ULN)) have significant liver inflammation, and those with ALT ≤2 ULN have no or mild liver inflammation. Therefore, guidelines for the management of chronic HBV infection suggested that ALT >2 ULN as one of the indications for antiviral therapy^4–6^. However, liver inflammatory activity grade is not always well correlated with ALT. Previous studies revealed that significant liver inflammation might be found in 20–34% of chronic HBV infection patients with detectable HBV DNA and persistently normal ALT (PNALT)^8–10^. Another study found 5.7% of chronic HBV infection patients with undetectable HBV DNA and PNALT had significant liver inflammation^11^. Obviously, using ALT to diagnose liver inflammation may miss a certain proportion of patients who have significant liver inflammation.

New noninvasive methods for the diagnosis of significant liver inflammation are needed urgently, especially in chronic HBV infection patients with detectable HBV DNA and PNALT. In this study, we aimed to develop a diagnostic algorithm to predict significant liver inflammation in chronic HBV infection patients with detectable HBV DNA and PNALT.

## Methods

### Study population

**Figure 1:**
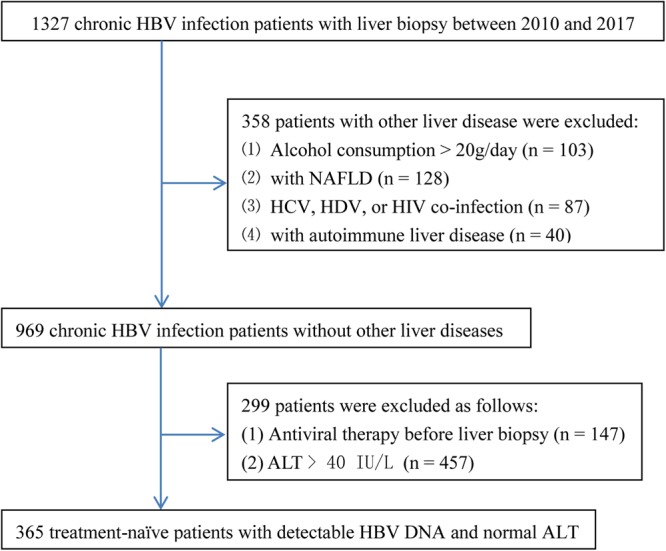
Flow diagram of the training set population. HBV, hepatitis B virus; NAFLD, non-alcoholic fatty liver disease; HCV, hepatitis C virus; HDV, hepatitis D virus; HIV, human immunodeficiency virus; ALT, alanine transaminase.

For this study, 1327 consecutive chronic HBV infection patients, who underwent liver biopsies at Shanghai Public Health Clinical Center, China, between January 2010 and January 2017, were retrospectively recruited. Chronic HBV infection was defined as the persistent presence of HBsAg for at least 6 months. Patients were excluded from this study for the following reasons: (1) alcohol consumption >20 g/day (n = 103); (2) with non-alcoholic fatty liver disease (n = 128); (3) co-infection with hepatitis C virus, hepatitis D virus, or HIV (n = 87); (4) with autoimmune liver disease (n = 40); (5) antiviral therapy before liver biopsy (n = 147); (6) ALT > ULN defined as 40 IU/L (n = 457). Finally, 365 treatment-naïve chronic HBV infection patients with detectable HBV DNA and PNALT (defined as normal ALT measured on at least three occasions at intervals of more than 2 months apart over a period of 12 or more months before liver biopsy) were included. Figure 1 summarized the flow diagram of the training set population.

**Figure 2:**
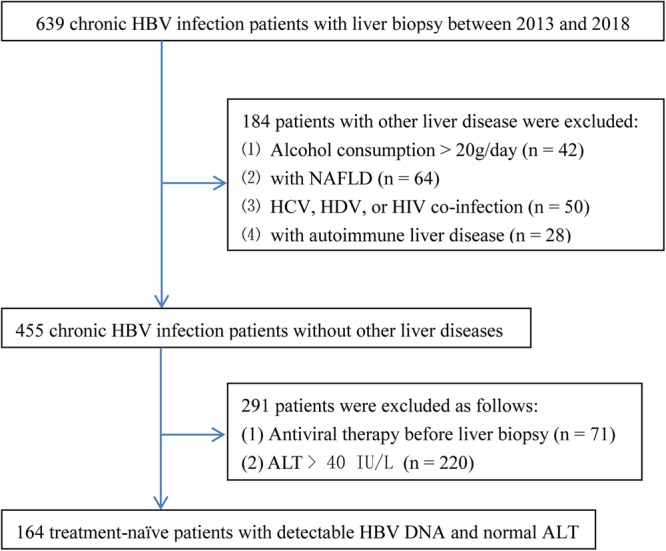
Flow diagram of the validation set population. HBV, hepatitis B virus; NAFLD, non-alcoholic fatty liver disease; HCV, hepatitis C virus; HDV, hepatitis D virus; HIV, human immunodeficiency virus; ALT, alanine transaminase.

One external validation set of treatment-naïve chronic HBV infection patients with detectable HBV DNA and PNALT (n = 164) who underwent liver biopsies at Ruian people’s hospital, Zhejiang, China, between January 2013 and May 2018, were recruited to assess the diagnostic performance of the novel diagnostic algorithm. The inclusion and exclusion criteria used were the same as those used for the patients who were enrolled in the training set. Figure 2 summarized the flow diagram of the validation set population.

All patients signed the informed consent before liver biopsy, and all clinical procedures were in accordance with the Helsinki declaration. The ethics committee of Shanghai Public Health Clinical Center approved the study protocol, and experiments, including any relevant details. All experiments were performed in accordance with relevant guidelines and regulations.

### Liver histology evaluation

Percutaneous liver biopsies were performed using a 16-G disposable needle under the guidance of ultrasound. The biopsy specimens were fixed with 10% formalin, embedded in paraffin, and stained with hematoxylin and eosin, Masson trichrome, and reticular fibre staining. A minimum of 15 mm of liver tissue with at least 6 portal tracts is considered sufficient for liver histological scoring. All biopsy samples were interpreted by two pathologists. In case of discrepancies, the biopsy samples were reviewed by a third highly experienced liver pathologist. Liver inflammation was divided into four stages according to the METAVIR scoring system^12^: A0, none inflammation; A1, mild inflammation (focal, few portal areas); A2, moderate inflammation (most portal areas, and extended to beyond the portal areas); and A3, severe inflammation (significant confluent necrosis and bridging necrosis). Significant liver inflammation was defined as inflammation stage ≥A2.

### Routine laboratory tests

Fasting blood samples were obtained, and routine laboratory tests were performed before liver biopsies. The serological markers of HBV were detected with enzyme-linked immune-sorbent assay kits (ARCHITECT i2000 SR; Abbott, Wiesbaden, Germany). The biochemical parameters including ALT, aspartate transaminase (AST), and gamma-glutamyl transpeptidase (GGT) were measured by a biochemistry analyzer (7600 Series; Hitachi, Tokyo, Japan). Platelet counts were detected with a hematology analyzer (XT-2000i, Sysmex, Kobe, Japan). HBV DNA was tested by real-time PCR with a lower limit of detection of 500 IU/mL.

### Statistical analysis

In order to identify predictors of significant liver inflammation, univariable regression analysis was performed for age, sex, HBeAg, HBV DNA, ALT, AST, alkaline phosphatase, GGT, total bilirubin, albumin, globulin, and platelet count. Multiple regression analysis was performed then by including the predictors associated with significant liver inflammation in the univariable regression analyses (*p* < 0.05). The final prediction model was selected using the β coefficients of the multivariate logistic regression analysis. The diagnostic accuracy was estimated using the area under the receiver operating characteristic curve (AUROC), and compared using the Delong test^13^. Three sets of cut-offs were calculated: (1) sensitivity ≥90%, (2) specificity ≥90%, or (3) Maximizing Youden’s index (balance between sensitivity and specificity). All significance tests were two-tailed, and *p* < 0.05 was considered statistically significant. All statistical analyses were performed using the SPSS software version 18.0 (IBM, Armonk, NY, USA) and MedCalc Statistical Software version 16.1 (MedCalc Software bvba, Ostend, Belgium). This study was reported in accordance with the Standards for Reporting of Diagnostic Accuracy (STARD)^14^.

## Results

### Baseline characteristics

The baseline characteristics of study population were shown in Table 1. In the training set, the majority of patients were male (53.2%), HBeAg positive (60.8%), and middle-aged (median, 36 years). The median HBV DNA, ALT, AST, alkaline phosphatase, GGT, total bilirubin, albumin, and globulin was 5.1 log10 copies/ml (IQR = 4.0–7.5), 27 IU/L (IQR = 20–32), 24 IU/L (IQR = 20–29), 70 IU/L (IQR = 58–82), 18 IU/L (IQR = 13–28), 13 umol/L (IQR = 9–19), 44 g/L (IQR = 42–46), and 30 g/L (IQR = 28–32), respectively, in the training set. No significant differences were found in baseline characteristics between the training and validation sets, except GGT (18 *vs* 15 IU/L, *p* < 0.001) and platelet count (172 *vs* 196 10^9^/L, *p* < 0.001). The prevalence of significant liver inflammation is 20.8% in the training set, and 20.1% in the validation set.

**Table 1:** Baseline characteristics of the study population.

Characteristics	Training set (n = 365)	Validation set (n = 164)	P value
Age (years)	36 (28–42)	38 (30–44)	0.079
Male gender, n (%)	194 (53.2%)	92 (56.1%)	0.529
HBeAg positive, n (%)	222 (60.8%)	106 (64.6%)	0.403
HBV DNA (log10 copies/ml)	5.1 (4.0–7.5)	6.0 (4.0–7.5)	0.887
ALT (IU/L)	27 (20–32)	28 (21–34)	0.058
AST (IU/L)	24 (20–29)	23 (21–27)	0.379
Alkaline phosphatase (IU/L)	70 (58–82)	70 (60–80)	0.843
GGT (IU/L)	18 (13–28)	15 (11–23)	<0.001
Total bilirubin (umol/L)	13 (9–19)	12 (10–16)	0.063
Albumin (g/L)	44 (42–46)	45 (41–48)	0.073
Globulin (g/L)	30 (28–32)	31 (27–34)	0.189
Platelet count (10^9^/L)	172 ± 55	196 ± 53	<0.001
Significant liver inflammation	76 (20.8%)	33 (20.1%)	0.854
Liver Inflammation stage			
A0	101 (27.7%)	34 (20.7%)	0.090
A1	188 (51.5%)	97 (59.1%)	0.103
A2	47 (12.9%)	22 (13.4%)	0.865
A3	29 (7.9%)	11 (6.7%)	0.618
Liver fibrosis stage			
F0	64 (17.5%)	26 (15.8%)	0.634
F1	211 (57.8%)	90 (54.9%)	0.529
F2	45 (12.3%)	23 (14.0%)	0.590
F3	25 (6.8%)	12 (7.3%)	0.845
F4	20 (5.4%)	13 (7.9%)	0.282

ALT, alanine transaminase; AST, aspartate transaminase; GGT, gamma-glutamyl transpeptidase.

### A novel diagnostic algorithm for significant liver inflammation

By univariate and multivariate regression analysis, age (OR = 1.049, *p* = 0.004), ALT (OR = 1.079, *p* = 0.002), GGT (OR = 1.031, *p* < 0.001), and platelet count (OR = 0.992, *p* = 0.017) were identified as the independent predictors of significant liver inflammation (Table 2). The four independent predictors were transformed into ordinal variables according to the thresholds corresponding to 33% and 66% prevalence for significant liver inflammation. The β coefficients of the multivariate analysis were used to determine a novel diagnostic algorithm: the AAGP algorithm. The ALT was capped at four points, to keep ALT from weighing too heavily in the AAGP algorithm. Finally, the AAGP algorithm is the sum of the scores from age, ALT, GGT, and platelet count (Table 3).

**Table 2:** The independent predictors of significant liver inflammation in the training set.

	Univariate analysis		Multivariate analysis	
	OR (95% CI)	P value	OR (95% CI)	P value
Age (years)	1.073 (1.044-1.103)	<0.001	1.049 (1.015–1.083)	0.004
Male	1.566 (0.933–2.626)	0.089		
HBeAg positive	1.056 (0.628–1.775)	0.838		
HBV DNA (copies/ml)	0.970 (0.842–1.118)	0.676		
ALT (IU/L)	1.107 (1.064–1.151)	<0.001	1.079 (1.029–1.131)	0.002
AST (IU/L)	1.051 (1.025–1.078)	<0.001	0.995 (0.967–1.023)	0.710
Alkaline phosphatase (IU/L)	1.021 (1.010–1.032)	<0.001	1.007 (0.993–1.021)	0.326
GGT (IU/L)	1.048 (1.032–1.063)	<0.001	1.031 (1.013–1.049)	<0.001
Total bilirubin (umol/L)	1.016 (0.983–1.049)	0.357		
Albumin (g/L)	0.963 (0.907–1.023)	0.217		
Globulin (g/L)	1.012 (0.956–1.072)	0.672		
Platelet count (10^9^/L)	0.984 (0.979–0.990)	<0.001	0.992 (0.986–0.999)	0.017

ALT, alanine transaminase; AST, Aspartate transaminase; GGT, gamma-glutamyl-transpeptidase.

**Table 3:** The AAGP algorithm.

Item		Points
Age (years)	≤30	0
	30–40	2
	>40	3
ALT (IU/L)	≤20	0
	20–30	1
	>30	4
GGT (IU/L)	≤50	0
	>50	2
Platelet count (10^9^/L)	≤100	3
	100–200	1
	>200	0

The four independent predictors were transformed into ordinal variables according to the thresholds corresponding to 33% and 66% prevalence for significant liver inflammation. The β coefficients of the multivariate analysis were used to determine a novel diagnostic algorithm: the AAGP algorithm. The ALT was capped at four points, to keep ALT from weighing too heavily in the AAGP algorithm. Finally, the AAGP algorithm is the sum of the scores from age, ALT, GGT, and platelet count.

### Compare the AAGP algorithm, ALT, and AST

**Figure 3:**
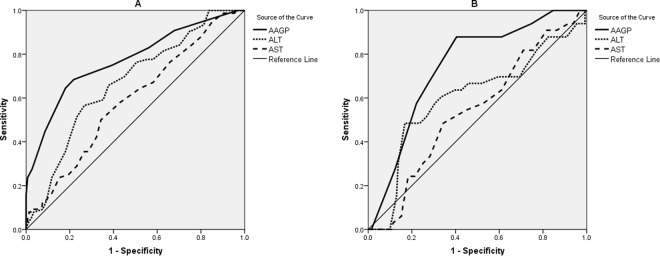
ROC curves of noninvasive tests in the training (**A**) and validation set (**B**). The AAGP algorithm is the sum of the scores obtained form age, ALT, GGT, and platelet count.

The ROC curves of the AAGP algorithm, ALT, and AST were shown in Fig. 3. In the training set, the AUROC of the AAGP algorithm was higher than that of ALT and AST to diagnose significant liver inflammation (0.77, 0.67, and 0.59, respectively, *p* < 0.001) (Table 4). In the validation set, the AUROC of AAGP algorithm was also higher than that of ALT and AST (0.75, 0.61, and 0.54, respectively, *p* < 0.001) (Table 4).

**Table 4:** Diagnostic performances of the AAGP algorithm for significant liver inflammation.

	Training set		Validation set	
	AUROC	(95% CI)	AUROC	(95% CI)
AAGP	0.77	(0.73–0.82)	0.75	(0.67–0.81)
ALT	0.67	(0.62–0.72)	0.61	(0.53–0.68)
AST	0.59	(0.54–0.64)	0.54	(0.46–0.62)
Comparison of AUROC				
AAGP vs ALT	*p* < 0.001		*p* < 0.001	
AAGP vs AST	*p* < 0.001		*p* < 0.001	

AAGP, a novel diagnostic algorithm for significant liver inflammation; ALT, alanine transaminase; AST, aspartate transaminase.

### Diagnostic thresholds of AAGP for significant liver inflammation

Diagnostic thresholds of the AAGP algorithm were presented in Table 5. Maximizing Youden’s index, the cut-off of AAGP was 5 for the diagnosis of significant liver inflammation (the sensitivity, specificity, positive predictive value (PPV), and negative predictive value (NPV) was 68%, 78%, 45%, and 90%, respectively). Using sensitivity ≥90%, the cut-off of the AAGP algorithm was 2 (the sensitivity, specificity, PPV, and NPV was 91%, 32%, 26%, and 93%, respectively). Using specificity ≥90%, the cut-off of AAGP was 8 for the diagnosis of significant liver inflammation (the sensitivity, specificity, PPV, and NPV was 45%, 91%, 58%, and 86%, respectively).

**Table 5:** Diagnostic thresholds of the AAGP algorithm.

	Cut-off	Se (%)	Sp (%)	PPV (%)	NPV (%)	+LR	−LR
Training set	5*	68	78	45	90	3.14	0.40
	2**	91	32	26	93	1.34	0.29
	8***	45	91	58	86	5.17	0.60
Validation set	5	67	73	38	90	2.43	0.46
	2	93	25	24	94	1.26	0.24
	8	27	88	36	83	2.23	0.83

AAGP, a novel diagnostic algorithm for significant liver inflammation; Cut-off* was obtained maximising Youden’s index; Cut-off** was obtained using sensitivity ≥90%; Cut-off*** was obtained using specificity ≥90%; Se, sensitivity; Sp, specificity; PPV, positive predictive value; NPV, negative predictive value; +LR, positive likelihood ratio; −LR, negative likelihood ratio.

## Discussion

Most often, physicians believe that ALT is the most sensitive non-invasive marker for the diagnosis of liver inflammation. However, significant liver inflammation might be found in 20–34% of chronic HBV infection patients with detectable HBV DNA and PNALT^8–10^, and 5.7% of chronic HBV infection patients with undetectable HBV DNA and PNALT^11^. In this study, using liver biopsy as the gold standard, we found that 20.1–20.8% of chronic HBV infection patients with detectable HBV DNA and PNALT have significant liver inflammation.

At present, non-invasive markers to predict significant liver inflammation in chronic HBV infection patients with PNALT is absence. In this study, we developed a new, simple, and inexpensive noninvasive algorithm, the AAGP, to identify patients with significant liver inflammation from chronic HBV infection patients with detectable HBV DNA and PNALT. We found that the AAGP algorithm have a higher diagnostic accuracy compared with ALT and AST for significant liver inflammation, which indicating the application prospects of the AAGP algorithm in chronic HBV infection patients with detectable HBV DNA and PNALT.

The AAGP algorithm is the sum of the scores obtained form age, ALT, GGT, and platelet count, which were commonly tested in patients with chronic HBV infection. The AAGP algorithm has several attractive features. First, AAGP provides a noninvasive evaluation of significant liver inflammation in chronic HBV infection patients with detectable HBV DNA and PNALT. Second, AAGP is inexpensive, and very easily calculated, which makes AAGP easier to use in clinical practice. Third, AAGP provided a clinically available method for the diagnosis of significant liver inflammation in resource-limited settings, where liver biopsy might be unavailable.

In this study, the AAGP algorithm ≥2 was more sensitive (91–93%) and less specific (25–32%) to diagnose significant liver inflammation (Table 5). This suggested AAGP ≥2 could be used for the screening of significant liver inflammation, and selection of candidates for liver biopsy, in chronic HBV infection patients with detectable HBV DNA and PNALT. AAGP ≥8 was more specific (88–91%) and less sensitive (27–45%) for the diagnosis of significant liver inflammation (Table 5). This suggested AAGP ≥8 could be used to diagnose significant liver inflammation, and avoiding partly liver biopsy in chronic HBV infection patients with detectable HBV DNA and PNALT.

In this study, the PPVs of AAGP algorithm were low (24–58%). In fact, the low PPVs were a common problem of noninvasive diagnostic methods. According to the WHO HBV guideline, the PPV was low (less than 50%) for all noninvasive liver fibrosis tests^15^. Based on the fact that the PPVs of AAGP were low, and many cases will be missed using AAGP solely to diagnose significant liver inflammation, we suggested that liver biopsy is still required to make a definite diagnosis for significant liver inflammation. Although the AAGP algorithm cannot replace liver biopsy, it can select the candidates for liver biopsy, avoid excessive liver biopsy, and narrow down the group which really needs liver biopsy.

In this study, ALT was identified as one of the independent predictors of significant liver inflammation (OR = 1.079, *p* = 0.002). This is consistent with a Chinese study, which identified ALT as an independent predictor of significant liver histological change in chronic HBV infection patients with PNALT (OR = 1.042, *p* < 0.001)^10^. A study from Taiwan also found ALT was associated with significant liver inflammation in HBV infection patients with normal ALT (OR = 1.82, *p* = 0.019)^16^. A study from Korea found, compared with the concentration <20 IU/L, the adjusted relative risks of significant liver inflammation for ALT concentration of 20–29 IU/L and 30–39 IU/L were 2.9 and 9.5 in men, and 3.8 and 6.6 in women, respectively^17^. The results that ALT is still associated with significant liver inflammation in patients with PNALT, indicated that, as the ULN of ALT, 40 IU/L might be higher for chronic HBV infection patients^6^.

Furthermore, our study found that GGT was an independent predictor of significant liver inflammation (OR = 1.031, *p* < 0.001). Previous studies have also shown that GGT is one of risk factors for significant liver inflammation in chronic HBV infection patients. For example, Myers *et al*. found that GGT was an independent predictor of significant liver inflammation in patients with CHB^18^. Yu *et al*. also found that GGT was an independent predictor of liver inflammation (OR = 1.007, *p* = 0.03) in CHB patients^19^. Wang *et al*. also found that GGT was an independent predictor of significant liver disease (OR = 1.03, *p* = 0.031) in CHB patients^20^.

In this study, there are some limitations. First, the retrospective design might have caused selective bias resulting in underestimated sensitivity and overestimated specificity of noninvasive diagnostic methods^21^. Therefore, prospective studies will be necessary to validate the clinical application of the AAGP algorithm. Second, this study excluded patients with antiviral therapy, other liver diseases, or elevated ALT. Consequently, the diagnostic value of the AAGP algorithm is unclear in chronic HBV infection patients with above conditions. Third, we have not provided an algorithm to discriminate significant fibrosis with normal ALT, which is more important in the clinical settings. Using this cohort, we had tried to develop a novel algorithm to discriminate significant fibrosis with normal ALT. Unfortunately, we did not find a novel algorithm, which have higher performance than the existing noninvasive fibrosis tests (APRI, FIB-4, GPR, and FibroScan), to diagnose significant fibrosis in chronic HBV infection patients with detectable HBV DNA and PNALT.

In conclusion, significant liver inflammation was found in 20.1–20.8% of chronic HBV infection patients with detectable HBV DNA and PNALT. The AAGP algorithm is a new, simple, noninvasive method, which can discriminate patients having significant liver inflammation in chronic HBV infection patients with detectable HBV DNA and PNALT. The purpose of the AAGP algorithm is to be used by physician to identify patients with significant liver inflammation who require further evaluation with liver biopsy or should be considered for antiviral therapy. Although the AAG algorithm cannot replace liver biopsy, it can select the candidates for liver biopsy, avoid excessive liver biopsy, and narrow down the group which really needs liver biopsy.
